# Correction: Sharma et al. Microbial Biofilm: A Review on Formation, Infection, Antibiotic Resistance, Control Measures, and Innovative Treatment. *Microorganisms* 2023, *11*, 1614

**DOI:** 10.3390/microorganisms12101961

**Published:** 2024-09-27

**Authors:** Satish Sharma, James Mohler, Supriya D. Mahajan, Stanley A. Schwartz, Liana Bruggemann, Ravikumar Aalinkeel

**Affiliations:** 1 Department of Urology, Jacobs School of Medicine and Biomedical Sciences, University at Buffalo, Buffalo, NY 14260, USA; ss466@buffalo.edu (S.S.); sasimmun@buffalo.edu (S.A.S.); 2 Department of Urology, Roswell Park Comprehensive Cancer Center, Buffalo, NY 14203, USA; james.mohler@roswellpark.org; 3 Department of Medicine, Division of Allergy, Immunology, and Rheumatology, Jacobs School of Medicine and Biomedical Sciences, University at Buffalo, Buffalo, NY 14203, USA; smahajan@buffalo.edu; 4 Department of Medicine, VA Western New York Healthcare System, Buffalo, NY 14215, USA; 5 Department of Biomedical Informatics, University at Buffalo, Buffalo, NY 14260, USA; lianabru@buffalo.edu

In the original publication [1], there was a missing reference to Figure 4 and Figure 5 as published. The revision substantially altered Figure 4 and Figure 5. Additionally, the legends for the corresponding Figures have been revised whereby an attribution has been added to the Figure legends mentioning the original article with the figure adaptation. The corrected Figures appear below. 

The authors state that the scientific conclusions are unaffected. This correction was approved by the Academic Editor. The original publication has also been updated.

## Figures and Tables

**Figure 4:**
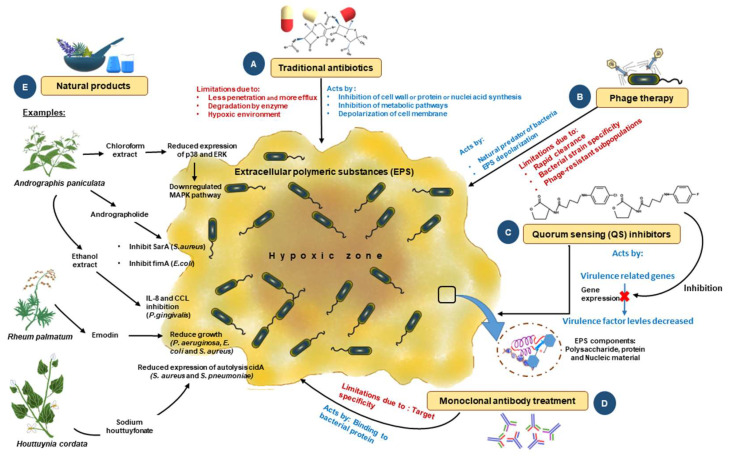
Traditional and novel methods to control biofilms: To fight infections ensuing from biofilms, numerous methods have been developed from different aspects; some are conventional, and some are novel. (**A**) Use of traditional antibiotics in the early stage; however, this method has a high failure rate due to poor penetration and a lack of action due to hypoxia. (**B**) Phage therapy has been used as an alternate approach for controlling biofilm formation. This method works by depolarizing the EPS to disrupt the biofilm. This method also has multiple limitations, including resistance, clearance by the host immune system, and the fact that it is specific only to certain strains of bacteria. (**C**) Novel methods of biofilm disruption, such as QS system inhibitors, which interfere with the microbial communication mechanism based on molecular signatures, are also being used. (**D)** Newer methods, such as antibody-based therapy against biofilms, are being tried in preclinical models that target several biofilms, but are limited in their success due to poor target specificity and infusion reactions. (**E**) Natural-product-based therapy is another conventional method used. The products used here are either crude extracts or purified compounds. Biologically active compounds showing antibacterial activity are extracted, purified, and successfully evaluated using clinical and pre-clinical models. Such extracts include chloroform, ethanol, and methyl ester. These extracts inhibit biofilms through various mechanisms ranging from the inhibition of a critical enzyme involved in the growth of bacteria to repression of the gene expression of multiple genes required for bacterial growth and development. This figure is adapted and modified from Mirghani et al. 2022 (ref [212]) in AIMS Microbiology (doi: 10.3934/microbiol.2022019. PMID: 36317001; PMCID: PMC9576500. https://www.ncbi.nlm.nih.gov/pmc/articles/PMC9576500/ (accessed on 1 April 2023)), an open-access article, and is reproduced under the Creative Commons BY 4.0 license.

**Figure 5:**
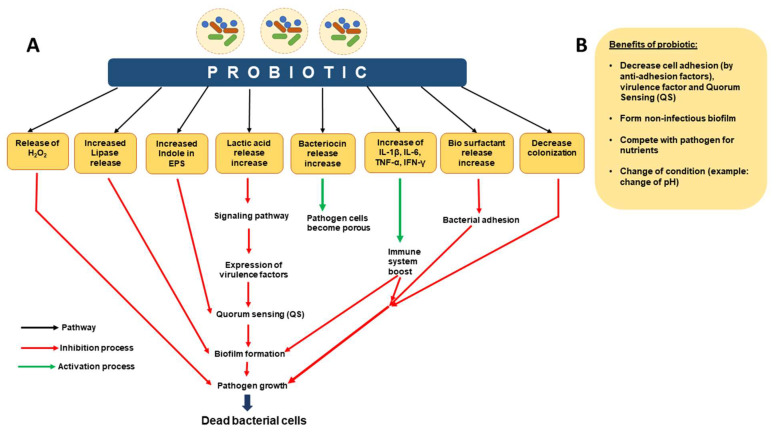
Probiotic methods to control biofilms. Probiotics pathogenic biofilm inhibition occurring in different ways. (**A**) Mechanism of action of probiotics. (**B**) Benefits of probiotics. This figure is adapted and modified from Mirghani et al. 2022 (ref [212]) in AIMS Microbiology (doi: 10.3934/microbiol.2022019. PMID: 36317001; PMCID: PMC9576500. https://www.ncbi.nlm.nih.gov/pmc/articles/PMC9576500/), an open-access article, and is reproduced under the Creative Commons BY 4.0 license.
